# A Case of Acute Adrenal Insufficiency: A Rare but Possible Cause of Hypercalcemia

**DOI:** 10.7759/cureus.24752

**Published:** 2022-05-05

**Authors:** Hayat Aynaou, Houda Salhi, Hanan El Ouahabi

**Affiliations:** 1 Department of Endocrinology, Diabetology, Metabolic Diseases and Nutrition, Hassan II University Hospital, Fez, MAR

**Keywords:** rehydration, hydrocortisone, unusual association, adrenal insufficiency, hypercalcemia

## Abstract

A 30-year-old woman presented with a 15-day history of epigastric pain, nausea, vomiting, asthenia, and weight loss. On admission, hypercalcemia was reported with a negative etiologic workup (including no primary hyperparathyroidism, malignancy, or vitamin D toxicity). Fluid replacement did not improve her hypercalcemia. We performed a blood ionogram and assessed the adrenocortical function, which showed that her serum cortisol was decreased, her adrenocorticotropic hormone (ACTH) was elevated, and anti-21 hydroxylase antibodies were positive. We established the diagnosis of autoimmune primary acute adrenal insufficiency. The patient was treated with hydrocortisone. Shortly after initiating the treatment, her serum calcium levels returned to normal and her symptoms improved.

This case report highlights the fact that even though adrenal insufficiency is an uncommon etiology of hypercalcemia, it should not be totally ignored, especially since hypercalcemia can sometimes be indicative of adrenal impairment.

## Introduction

Hypercalcemia is a condition commonly encountered in medical practice. The clinical signs of hypercalcemia are myriad and can sometimes be seriously misleading, which can prove to be life-threatening. Clinical manifestations of the condition depend on the extent of calcium elevation and especially the rapidity of its rise. In general, malignancy accounts for 70% of cases of hypercalcemia, followed by primary hyperparathyroidism in 20% of cases, and other causes in 10% [e.g., granulomatosis, drugs (vitamin D toxicity, thiazide diuretic), hyperthyroidism, milk-alkali syndrome, and immobilization] [1-2].

Adrenal insufficiency is a recognized but rare cause of hypercalcemia [2]. It can arise from a primary adrenal disorder, secondary to adrenocorticotropic hormone (ACTH) deficiency, or due to the suppression of ACTH by exogenous glucocorticoids. The prevalence of hypercalcemia at the time of Addison's disease diagnosis is estimated to be ~5.5-6.0% [3]. We report the case of a patient with autoimmune primary adrenal insufficiency who presented with hypercalcemia that responded to glucocorticoid replacement therapy.

## Case presentation

The patient was a 30-year-old woman with no family history of autoimmune pathologies, who had been followed up for Hashimoto's thyroiditis. She presented to our service with a complaint of vomiting for the past two weeks, epigastric pain, profound physical and psychological asthenia, anorexia, and a weight loss of 13 kg. She had regular menstrual cycles. Physical examination revealed the following findings - height: 153 cm, bodyweight: 50 kg, blood pressure: 80/50 mmHg, heart rate: 90 beats per minute, and respiratory rate: 20 breaths per minute, with a body temperature of 37 °C. Her skin (notably looking for vitiligo), pulmonary, cardiac, abdominal, and neurologic examinations were unremarkable.

A biochemical profile (Table 1) revealed plasma total calcium of 144 mg/l (normal range: 84-105) and albumin of 39 g/1 (normal range: 37-52). The etiological evaluation was negative, with no history of drug or calcium intake or vitamin A or vitamin D toxicity. She had no signs that indicated endocrine pathologies (notably no acromegaly), primary hyperparathyroidism, or malignancy [parathyroid hormone (PTH) was low at <4 pg/ml and vitamin D was at 9.1 ng/ml; serum protein electrophoresis and cervicothoracoabdominopelvic CT showed no abnormalities]. Other biochemistry investigations revealed hyponatremia with a sodium level of 126 meq/l (normal range: 135-145), hyperkalemia with potassium at 5.2 meq/l (3.4-4.5), functional kidney failure, and a normal blood count. In light of the results of this ionogram, acute adrenal insufficiency was suspected and an evaluation of the adrenal function was completed. This assessment showed that her serum cortisol level was 1 μg/dL, ACTH was 186.6 pmol/l (normal range: 1.1-13.2), and anti-21 hydroxylase antibody level was >100 u/ml (positive if >0.5 u/ml), and hence the diagnosis of autoimmune primary adrenal insufficiency was retained. The rest of the biological assessment was as follows: thyroid-stimulating hormone (TSH) at 9.14 uIU/mL (normal range: 0.30-5.0), FT4 at 0.77 ng/dl (normal range: 0.7-1.55), and positive anti-thyroperoxidase antibodies at 1000 IU/ml. Her fasting blood sugar was 0.98 g/l with glycated hemoglobin at 5.4%. Her blood count was normal, with no macrocytic anemia.

After the initiation of rehydration and hydrocortisone therapy, her serum calcium level returned to normal and her symptoms, including epigastric pain and vomiting, improved. The rest of the assessment also revealed normal findings, especially sodium, potassium, and PTH.

Acute adrenal insufficiency was considered the cause of the patient's hypercalcemia, especially after excluding other more common diseases and the normalization of calcium levels after the initiation of hormone replacement therapy for adrenal insufficiency.

**Table 1:** Laboratory data on admission and after glucocorticoid replacement therapy PTH: parathyroid hormone; ACTH: adrenocorticotropic hormone

Variables	On admission	After glucocorticoid replacement therapy	References ranges
Total calcium	144	96	84-105 mg/l
Albumin	39	39	37-52 g/l
Phosphorus	30	29	25-45 mg/l
PTH	4	14	10-65 pg/ml
Sodium	129	140	135-145 meq/l
Potassium	5.2	4	3.4-4.5 meq/l
8-hour cortisol	1	-	5-18 μg/dL
ACTH	186.6	-	1.1-13.2 pmol/l
Anti-21 hydroxylase antibodies	>100	-	<0.5 u/ml

## Discussion

Adrenal insufficiency is a rare cause of mild to moderate hypercalcemia [4-5], and the prevalence of this kind of hypercalcemia is variable depending on the type of adrenal insufficiency. The rate of prevalence of hypercalcemia is unknown in secondary adrenal insufficiency, while it is approximately 5.5-6.0% in the case of Addison's disease [3], as with our patient.

Hypercalcemia in adrenal insufficiency is usually mild to moderate [4-5]. In a recent study, 53% of 212 intensive care unit patients were diagnosed with latent adrenal insufficiency, but none of these patients developed overt hypercalcemia [6]. The unusual feature of this case was the severe hypercalcemia. Primary hyperparathyroidism was excluded on account of the suppressed serum PTH levels. Hyperthyroidism, vitamin D toxicity, and malignancy were also excluded (protein electrophoresis and cervicothoracoabdominal CT scan were normal, and vitamin D level was low).

Clinical signs of hypotension and abnormal serum electrolytes (hyponatremia and hyperkalemia) provided clues to the cause of the hypercalcemia, and it was the eight-hour cortisol that ultimately confirmed the diagnosis of adrenal insufficiency. The patient received rehydration and glucocorticoid replacement, which led to the normalization of her serum calcium, and this provided further confirmation that adrenal insufficiency was the cause of her hypercalcemia.

The mechanism of hypercalcemia in adrenal insufficiency has not been fully elucidated, but it is likely to be multifactorial. Firstly, adrenal insufficiency causes hypovolemia and thus reduces glomerular filtration. The decrease in glomerular filtration leads to a reduction in the amount of filtered calcium and an increase in the renal reabsorption of calcium in the proximal tubule [7-8]. Volume repletion has a beneficial effect on renal filtrated calcium, whereas increased calcium reabsorption in the proximal tubules is not reversed by volume repletion, and normalizes only after the replacement of glucocorticoids [9]. The use of hydrocortisone leads to the retention of sodium and the reduction in the reabsorption of calcium in the proximal tubule. Secondly, the enzymatic activity of 1-alpha-hydroxylase may be increased in adrenal insufficiency. The 1-alpha-hydroxylase is a renal enzyme responsible for the conversion of calcidiol to calcitriol, leading to increased intestinal absorption of calcium and consequently to hypercalcemia [10]. The third mechanism to explain the presence of hypercalcemia in adrenal insufficiency is an increase in calcium efflux of bone into the circulation [9]. The presence of glucocorticoid receptors on the bone cells indicates that physiological quantities of glucocorticoid hormones are necessary for the acquisition and preservation of the differentiated state of osteoblasts [11]. However, it is not clear whether glucocorticoid deficiency is the only factor responsible for increased calcium efflux from the bone.

Based on this case report, we recommend that clinicians consider adrenal insufficiency as a cause of hypercalcemia, obviously after excluding the other most frequent etiologies.

## Conclusions

Hypercalcemia is a fairly common medical condition. Although primary hyperparathyroidism and malignancy are the most common causes of hypercalcemia, adrenal insufficiency is also a rare but important cause of hypercalcemia. Since not all cases of adrenal insufficiency are accompanied by hypercalcemia, insufficiency is not often readily considered an etiology of hypercalcemia. In our case, adrenal insufficiency as the cause of hypercalcemia was suspected, after excluding the other most common diseases, and was confirmed based on the normalization of serum calcium after hormonal treatment with glucocorticoids.

Even though adrenal insufficiency is an exceedingly rare etiology of hypercalcemia, it should not be completely disregarded by clinicians.

## References

[1] Ziegler R (2001). Hypercalcemic crisis. J Am Soc Nephrol.

[2] Jacobs TP, Bilezikian JP (2005). Clinical review: rare causes of hypercalcemia. J Clin Endocrinol Metab.

[3] Nerup J (1974). Addison's disease--clinical studies. A report fo 108 cases. Acta Endocrinol (Copenh).

[4] Leeksma CH, De Graeff J, De Cock J (1957). Hypercalcaemia in adrenal insufficiency. Acta Med Scand.

[5] Downie WW, Gunn A, Paterson CR, Howie GF (1977). Hypercalcaemic crisis as presentation of Addison's disease. Br Med J.

[6] Kromah F, Tyroch A, McLean S, Hughes H, Flavin N, Lee S (2011). Relative adrenal insufficiency in the critical care setting: debunking the classic myth. World J Surg.

[7] Bhatti RS, Flynn MD (2012). Adrenal insufficiency secondary to inappropriate oral administration of topical exogenous steroids presenting with hypercalcaemia. BMJ Case Rep.

[8] Lee AS, Twigg SM (2015). Opioid-induced secondary adrenal insufficiency presenting as hypercalcaemia. Endocrinol Diabetes Metab Case Rep.

[9] Muls E, Bouillon R, Boelaert J, Lamberigts G, Van Imschoot S, Daneels R, De Moor P (1982). Etiology of hypercalcemia in a patient with Addison's disease. Calcif Tissue Int.

[10] Minisola S, Pepe J, Piemonte S, Cipriani C (2015). The diagnosis and management of hypercalcaemia. BMJ.

[11] Raisz LG, Kream BE (1983). Regulation of bone formation. N Engl J Med.

